# Supplementary material for: Anti-Ballistic Performance of PPTA/UHMWPE Laminates
Source: Polymers (Basel). 2023 May 12;15(10):2281. doi: 10.3390/polym15102281 (PMC10224376; doi:10.3390/polym15102281)
Supplement: Supplementary file 1 [file polymers-15-02281-s001.zip › polymers-2337050-supplementary.pdf]

Supporting information

Anti-ballistic performance of PPTA/UHMWPE laminates

Long Zhu [1], Weixiao Gao [1], Dmitriy A. Dikin [1], Simona Percec [2], Fei Ren [1,*]

1 Department of Mechanical Engineering, Temple University, Philadelphia, PA 19122, USA; long.zhu@temple.edu (L.Z.); weixiao.gao@temple.edu (W.G.); ddikin@temple.edu (D.A.D.)
2 Temple Materials Institute, Temple University, Philadelphia, PA 19122, USA; simona.percec@temple.edu
* Correspondence: renfei@temple.edu

Table S1. Comparison of the properties of PPTA fabric and UHMWPE film.

Properties	PPTA fabric	UHMWPE film
Density (g cm^{-3})	1.44	0.94
Tensile modulus (GPa)	59 - 124	0.2 - 1.2
Tensile strength (MPa)	2760	20 - 40
Coefficient of thermal expansion ($\times 10^{-6}$ K^{-1})	-2 Along Axis	130 - 200
Specific heat (J K^{-1} kg^{-1})	1400	1900
Thermal conductivity (W m^{-1} K^{-1})	0.04 @23°C	0.42 – 0.51 @ 23°C
Upper working temperature (C)	180 - 245	55 - 95

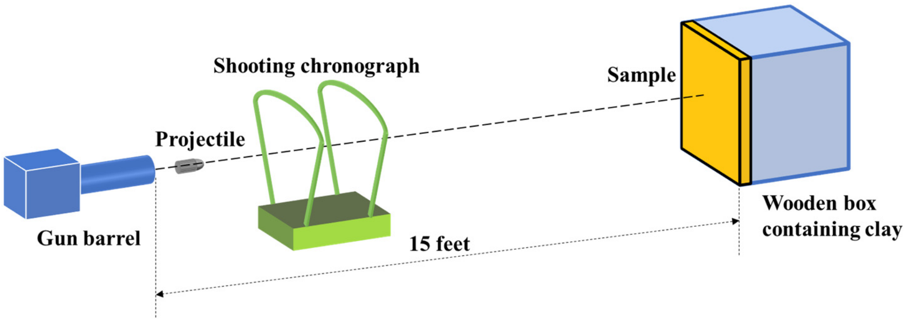

Figure S1. Schematic of the experimental setup for the ballistic testing.

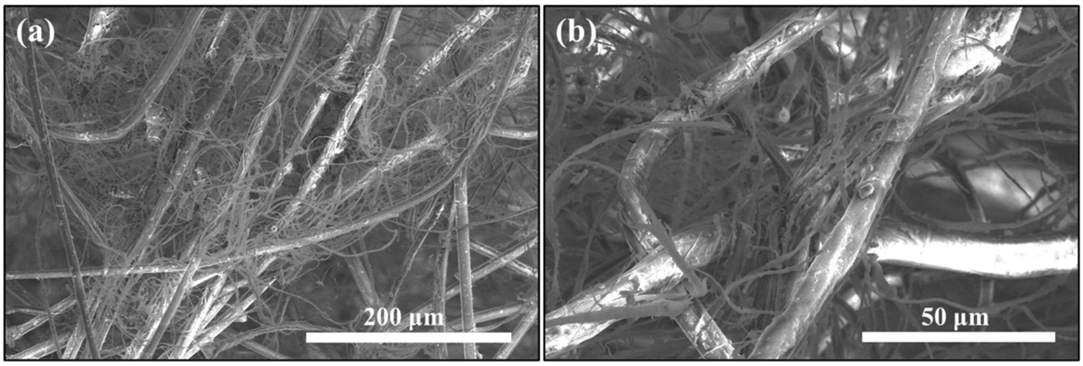

Figure S2. SEM images of the PPTA fibers captured on the exit side of the laminate sample (u)PE(9)-(5)KF(8) around a completely penetrated hole.
